# “Like something supernatural in your house”: an interpretative phenomenological analysis to explore the experiences and psychological challenges of parents raising children with autism spectrum disorder

**DOI:** 10.1186/s40359-025-03057-5

**Published:** 2025-07-01

**Authors:** Tetiana Yevtushok, Dominic Petronzi

**Affiliations:** 1 University of Derby, Kedleston Road, Derby, Derbyshire, DE22 1GB UK; 2 School of Psychology, University of Derby, Derby, UK

**Keywords:** ASD, Qualitative research, Parental experiences, Growth through adversity

## Abstract

**Background:**

Parents raising children with ASD face profound psychological challenges. While existing research predominantly focuses on parental distress, opportunities for growth and transformation remain underexplored. Addressing these gaps, this study employs an existential framework and Interpretative Phenomenological Analysis to provide a nuanced understanding of parental psychological experiences, emphasizing how they navigate and transcend the challenges of raising a child with ASD.

**Methods:**

Semi-structured interviews were conducted with four parents (three mothers and one father) of children diagnosed with ASD for at least three years. Participants ranged in age from 31 to 50 years, while their children, all male, were aged 11 to 22 years. The participants represented diverse geographic backgrounds, including Canada, the United Arab Emirates, the United Kingdom, and the United States.

**Results:**

The analysis identified two superordinate themes: “*From Existential Crisis to Enlightenment*” and “*Transcending Challenging Experiences*.” Participants initially experienced an emotional breakdown following their child’s diagnosis, marked by grief and uncertainty. Over time, they transitioned from despair to faith, cultivating acceptance and spiritual beliefs as essential coping mechanisms. They also confronted significant emotional challenges, including guilt, fear of death, and communication struggles, ultimately fostering resilience and forming transcendent relationships with their children.

**Conclusions:**

The findings illuminate the dual dimensions of the parental experience, encompassing both distress and growth. This study offers deeper insights into the emotional and existential aspects of parenting a child with ASD and underscores the need for tailored interventions to support parents in navigating these transformative journeys.

**Supplementary Information:**

The online version contains supplementary material available at 10.1186/s40359-025-03057-5.

## Introduction

Existential philosophy emphasises the transformative potential of adversity, where individuals face a choice between despair and faith. This existential perspective is particularly relevant when applied to the experiences of parents raising children with Autism Spectrum Disorder (ASD). Beyond distress, this perspective highlights how navigating emotional challenges can catalyse growth and meaningful change. By framing parental experiences within this lens, the current study seeks to move beyond conventional perceptions of autism-related challenges, exploring their deeper transformative potential.

ASD is a complex neurodevelopmental condition marked by persistent patterns in behaviour, interests, and activities [1]. Diagnostic criteria for ASD as outlined in the DSM-5 include deficits in ‘social-emotional reciprocity,’ ‘non-verbal communication,’ and challenges in ‘developing, maintaining, and understanding relationships’ [1]. Recent prevalence data indicate that 1 in 36 eight-year-old children in the United States were estimated to have ASD in 2020 [2], underscoring the widespread impact of this diagnosis on families and society at large.

Research has consistently shown that parents of children with ASD experience significantly higher levels of stress than parents of children with other developmental disorders [3, 4]. Parenting a child with ASD is associated with a range of psychological challenges, including higher rates of depression, anxiety, and PTSD [5–7]. The stress of caring for a child with ASD affects parents’ well-being [4] and is often compounded by the use of avoidant coping strategies [8] that can negatively affect both parental well-being and family dynamics. Dysfunctional coping strategies have been linked to high rates of marital discord and divorce among parents of children with ASD [9]. Emotion-focused and avoidant coping strategies correlate with heightened stress and burden [10–12].

Parents of children with ASD frequently encounter challenging experiences, including struggles with communication barriers, social isolation, feelings of guilt, shame, and fears surrounding death [13–15], that may drive parents toward avoidant and emotion-focused coping, which can influence not only their mental health but also their children’s behaviour. Studies show that parents’ moods and emotional states directly impact their autistic children’s behaviours [16, 17]. A thematic analysis of interviews with autistic adults found that a perceived lack of acceptance increased their engagement in stereotyped and repetitive movements [18]. Di Renzo et al. [19] found that greater parental insightfulness and acceptance of the autism diagnosis were associated with higher levels of attunement and sensitivity in caregiving. Positive and negative perceptions play a significant role, with positive perceptions linked to increased life satisfaction [20]. These findings suggest that some parents struggle to manage the intense emotions related to their child’s diagnosis, often resorting to avoidance, while others actively choose to confront these emotions, finding ways to cope and develop faith [21].

While much of the current research focuses on the psychological strain associated with ASD, relatively few studies explore the potential for growth and resilience among these parents. Research demonstrates that distress and growth often coexist [22], creating a “battlefield of emotions” where parents must navigate between despair and resilience. This dynamic aligns with [23, 24] theory of existential transformation, wherein individuals actively engage with boundary situations– such as guilt, death, uncertainty and struggle (including dissatisfaction with communication)– to transcend conventional norms and derive new meanings that foster growth, resilience and spirituality. Notably, studies show that parents with heightened spirituality often adapt more effectively to challenges [25]. Waizbard-Bartov et al. [26] highlight that parents of children with ASD experience personal growth, discovering spirituality, resilience and reevaluating life’s priorities. Spirituality has been linked to decreased depressive symptoms and increased satisfaction, self-esteem, and a sense of control among parents of children with ASD [25]. Spiritual training programs, including mindfulness practices, have shown positive effects on resilience in these parents [27].

Although studies demonstrate the development of spirituality as a source of support for parents [25–27] describing the observed phenomena (what is revealed), they frequently lack an in-depth interpretation of the processes involved (how these phenomena unfold). Existing research does not usually dive deeper into parents’ challenging emotional experiences to explain their meanings and transformative potential. For example, when spirituality is noted as a transformative experience for parents [25], the mechanisms driving this transformation remain unclear due to methodological limitations. A phenomenological approach, particularly Interpretative Phenomenological Analysis (IPA), is therefore well-suited to uncover how parents interpret and assign meaning to their emotionally intense experiences [28]. Understanding these processes could provide valuable insights into the pathway to growth and acceptance on an existential level, ultimately helping parents adapt.

In the context of ASD, authentic acceptance aligns with Sartre’s [29] notion of embracing existence as it is, rather than as it should be, reflecting the parental journey toward accepting their child’s diagnosis. This process involves recognizing and validating the child’s unique ways of perceiving and interacting with the world, including differences in sensory processing and communication. Acceptance, therefore, is not passive resignation [23] but an intentional, hopeful choice– one that reframes the perception of an autistic child from being “damaged” to being a whole and valuable human being. This existential transformation is accompanied by challenging emotions such as despair, guilt, shame, and fear of death, signalling an internal struggle with acceptance. However, existential thought posits that these emotions are inevitable and necessary for growth. Frankl [30] asserted that meaning arises from one’s response to unavoidable suffering, suggesting that these emotions serve as catalysts for self-exploration and transformation. By forcing individuals to question and transcend habitual norms, these emotional struggles pave the way for newfound meaning and what Jaspers [24] terms existential enlightenment. In this context, boundary situations foster growth through adversity, leading to a deeper appreciation for life, more meaningful relationships, greater personal resilience, shifted priorities, and enhanced existential and spiritual awareness [31]. This existential perspective was central to this study, providing a deeper understanding of how parents raising children with ASD navigate their lived experiences and find meaning in the face of profound challenges.

In summary, while existing research has shed light on the psychological difficulties faced by parents of children with ASD [5–7], it often lacks a comprehensive understanding of how they adapt to these challenges, make meaning of their experiences, and achieve growth through adversity. For example, Tsioka et al. (2022) [32] found evidence of post-traumatic growth among these parents, identifying cognitive reframing and depressive symptoms as significant predictors. However, the underlying processes by which parental challenges are transformed into growth remain under-explored, underscoring the need for further investigation into the mechanisms of meaning-making and existential adaptation. The current study thus aimed to explore the lived experiences of parents raising children with ASD, focusing on the emotional and relational challenges they encounter. By applying an existential framework, this research offered a more nuanced understanding of parental challenges and growth, potentially informing interventions that promote resilience, acceptance, and well-being among families affected by ASD.

## Method

### Design

The research utilised a qualitative approach that supports the exploration of insights into personal and subjective experiences. The reactive design facilitated interaction between participants and the researcher, enabling the collection of rich, meaningful narratives with the potential to inform future intervention design.

This study employed semi-structured interviews as the primary data collection method and Interpretative Phenomenological Analysis as the analytical methodology. These methods were chosen to capture the nuanced perspectives of parents and explore the complexities of their emotional and relational challenges in raising children with ASD. IPA, as outlined by Smith et al. [28], involves several key steps, including reading and re-reading transcripts, initial noting, developing emergent themes, searching for connections across emergent themes, analysing subsequent cases, and identifying patterns across cases. This rigorous process enables the identification of superordinate themes that encapsulate the essence of participants’ experiences. The analysis also acknowledges the interpretative role of the researcher, ensuring a deep and nuanced understanding of the data.

An inductive approach was used to analyse the qualitative data collected from interviews. This approach involves developing patterns, themes, and categories directly from the data rather than testing predefined hypotheses or theories [33]. Inductive analysis is particularly well-suited for exploratory research, where the aim is to generate detailed insights into participants’ lived experiences. By allowing themes to emerge organically, this method ensures that the findings remain closely grounded in the participants’ own words and perspectives, offering an authentic representation of their experiences [34]. Additionally, the study adopted a phenomenological philosophical approach [28] to explore individual lived experiences. IPA draws on phenomenological philosophy, emphasising the importance of understanding participants’ lived experiences from their own perspectives [35]. Central to this methodology is the double hermeneutic process, in which the researcher seeks to make sense of how participants interpret their own experiences [36]. IPA is also idiographic, focusing on the detailed examination of individual cases before identifying broader patterns across participants [28].

### Participants

The study employed a purposive sampling strategy to select parents raising children diagnosed with ASD for at least three years (*N* = 4; 3 females and 1 male). This sample size aligns with Interpretative Phenomenological Analysis, which prioritizes depth over breadth, allowing for an in-depth exploration of participants’ lived experiences while maintaining the feasibility of data collection and analysis [28].

IPA studies typically use 3 to 10 participants to allow for in-depth, idiographic analysis while maintaining methodological rigor [28]. IPA focuses on theoretical transferability rather than statistical generalizability [37]. A smaller sample facilitates prolonged engagement with the data, capturing nuanced meaning-making processes that larger samples may dilute [38].

The inclusion criteria were designed to ensure that participants had sufficient time to process, reflect on, and adapt to the challenges of raising a child with ASD, thereby providing rich, meaningful data for analysis. The age range of participants (31 to 50 years) and their children (11 to 22 years, all male) reflects a diverse range of caregiving experiences across different developmental stages.

Furthermore, the geographic diversity of the participants– representing Canada, the United Arab Emirates, the United Kingdom, and the United States– enhances the study’s ability to capture culturally and contextually varied perspectives on the existential and emotional dimensions of parenting a child with ASD. While the sample size is small, as is typical in IPA research, it is methodologically appropriate for generating deep, idiographic insights that can inform future research and intervention strategies.

### Materials

To conduct this study using IPA, a semi-structured interview guide comprising open-ended questions was developed to explore the lived experiences of parents raising children with ASD (see Supplementary File 1). The research questions were carefully crafted to align with the study’s aims and objectives and were informed by existing empirical findings. Research indicates that parents of children with ASD often experience a range of emotions following their child’s diagnosis, including initial shock and grief, followed by adaptation and acceptance [39]. For instance, the sample question, *“Can you describe your emotional journey since your child was diagnosed with ASD?”* was designed to capture this emotional evolution. Additionally, coping strategies and stress management, identified in the literature as crucial themes for parents of children with ASD who face elevated stress levels due to caregiving demands [10], were addressed through questions like, *“How do you typically cope with any stress associated with raising a child with ASD?”* These questions aimed to encourage participants to reflect deeply on their experiences, yielding rich qualitative data to support the IPA approach.

Additional research materials included Microsoft Forms to provide access to essential documents such as information sheets, consent forms, demographic questionnaires, and scheduling preferences. Virtual interviews were conducted and recorded via Microsoft Teams, leveraging its transcription functionality. The recorded interviews were securely stored on OneDrive.

### Procedure

Participants were recruited online via social media platforms. Permission to contact potential participants was obtained from group moderators. A research invitation was disseminated in relevant WhatsApp groups dedicated to parents of children with ASD. This invitation provided an overview of the study, including its purpose, procedures, inclusion and exclusion criteria, and a Microsoft Forms link for those interested in participating. Upon receiving consent, participants were contacted by the researcher to schedule interviews. The semi-structured interviews were conducted virtually using Microsoft Teams. All interviews were conducted in English, and fluency in English was an inclusion criterion for participation. Both audio and video recording were used during the interviews, with informed consent obtained in advance. The consent process included explicit permission for recording for research purposes, and participants were assured that recordings would be used solely for transcription and analysis. Each interview lasted between 60 and 80 min, providing ample time for participants to reflect and elaborate on their experiences.

The interviews were guided by a semi-structured question schedule, designed to explore themes such as emotional adaptation, coping strategies, social support, and the impact of raising a child with ASD on personal identity and relationships. The flexible nature of this format allowed the researcher to probe deeper into significant areas raised by participants while encouraging open-ended, detailed responses. For example, if a participant elaborated on coping strategies but had not yet addressed challenges with social support, the researcher sensitively redirected the conversation to ensure comprehensive coverage of all themes. This approach ensured that participants could fully express their experiences while maintaining focus on the core research questions.

To minimize the risk of overlooking relevant insights, each interview concluded with an open-ended question inviting participants to share any additional experiences or challenges not previously discussed. This final question aimed to capture any unique perspectives or unaddressed aspects of their journey. By offering this opportunity, the researcher ensured that the data collection was exhaustive, and participants felt their narratives were fully acknowledged.

Following the interviews, a detailed debrief form was emailed to participants, summarising the research, their rights, the withdrawal process, and additional resources for mental health support. Contact information for the researcher was also provided to address any follow-up questions or concerns.

The interviews were transcribed verbatim by the researcher. Transcription files were securely stored on the researcher’s OneDrive, adhering to strict confidentiality protocols. Once the transcriptions were finalised, the original audio and video recordings were deleted to further safeguard participant privacy.

### Analytical strategy

Data were analysed using Interpretative Phenomenological Analysis, a methodology well-suited for examining how individuals make sense of their lived experiences. The core analytic process was conducted by the first author, following the six-step framework outlined by Smith et al. [28]: reading and rereading transcripts, initial noting, developing emergent themes, searching for connections across emergent themes, analysing subsequent transcripts, and identifying patterns across transcripts. This systematic approach ensured thorough engagement with the data, yielding valuable insights into participants’ experiences.

The second author provided oversight by reviewing selected transcripts, extracts, and the narrative structure of the results section to ensure that the interpretation remained grounded in participants’ accounts and aligned with IPA principles. This collaborative approach supported reflexivity and analytic rigour.

During the initial phase, transcripts were read and reread to allow the researcher to fully immerse themselves in the data and develop an in-depth understanding of each participant’s narrative. This process involved closely engaging with the tone, structure, and flow of the interviews, facilitating an entry into the participant’s subjective world. Detailed notes were taken along three dimensions: descriptive (focusing on the content of what was said), linguistic (exploring the language and expression used), and conceptual (interpreting deeper meanings and connections). This multi-layered approach provided a comprehensive foundation for identifying emergent themes.

Emergent themes were derived by synthesising patterns observed in each participant’s narrative and framed in terms of their psychological and existential significance. These themes reflected both the explicit content, and the underlying meanings embedded in participants’ accounts. Using techniques such as abstraction (grouping related themes into broader categories) and contextualization (situating themes within the participant’s broader narrative), the researcher organized related themes into higher-order, superordinate categories. After analysing each case individually, shared themes were identified across cases, revealing superordinate themes such as Existential Crisis and Acceptance, Transcending Challenging Emotions, and Existential Transformation. This process balanced the idiographic focus on individual experiences with a nomothetic approach to uncover shared existential challenges and coping strategies among participants.

In the final stage, the researcher synthesised the findings into a coherent narrative that connected the superordinate themes to existential concepts. This interpretative phase emphasised how participants’ individual experiences reflected both personal meaning-making and universal existential dilemmas, as explored in the phenomenological and existential literature.

To ensure rigor and reflexivity, the researcher was mindful of her own experiences as a parent of a child with ASD, acknowledging this potential influence on interpretation. By engaging in IPA’s double hermeneutic process– interpreting participants’ interpretations of their experiences [36]– the researcher actively reflected on and mitigated potential biases. This reflexive approach enhanced the depth and authenticity of the analysis, ensuring that the findings remained rooted in participants’ perspectives while engaging critically with existential frameworks.

### Ethical considerations

To ensure confidentiality and ethics, the research was adhered to the British Psychological Society ethical guidelines [40]. The research was cleared through the university Research Ethics Committee (ETH2324-5379). Participants were provided with full study information and gave their informed consent to take part in the research and for their anonymised data to be used for publication purposes. To protect participant anonymity, pseudonyms were used in all transcripts and publications, and any potentially identifying information was removed or altered. Audio and video recordings were securely stored and deleted following transcription. Anonymised data were stored on a password-protected institutional account. The data in this research has not been used in previous works/published papers.

### Reflexivity

The researcher’s dual roles as a mother of a child with ASD and a practicing psychotherapist significantly informed the approach to this study. These lived and professional experiences provided a profound understanding of the emotional and practical challenges faced by parents raising children with ASD. This insight facilitated a sensitive and empathetic engagement with participants, fostering trust and enabling the collection of rich and detailed narratives.

However, the researcher was mindful of the potential influence of personal experiences and professional background on the research process, particularly during the interpretative stages. To mitigate this, ongoing reflexive practices were employed throughout the study. The researcher regularly documented reflections, critically examining areas where personal experiences might align with or diverge from the participants’ accounts. This process aimed to ensure that interpretations remained grounded in the data and were not unduly influenced by pre-existing assumptions.

The researcher’s psychotherapeutic training also contributed to creating a safe and supportive environment for participants to share their experiences. While this was a strength, the potential for over-identification with participants’ struggles was acknowledged. To address this, strategies such as critical self-reflection were utilised to maintain analytical objectivity.

By engaging in these reflexive practices, the researcher ensured that the study findings authentically represented the participants’ lived experiences. At the same time, the researcher’s unique perspective enriched the depth of interpretation, particularly within the existential framework that underpinned this research.

## Results

**Table 1:** Superordinate and subordinate themes, and participant convergence/divergence

Superordinate and Subordinate Themes	P1 (Julia)	P2 (Marta)	P3 (Peter)	P4 (Anastasia)
**From Existential Crisis to Enlightenment**				
- *Diagnosis as an Existential Crisis* - *Acceptance as a Form of Existential Enlightenment*	* *	* *	* *	* *
**Transcending Challenging Experiences**				
- *Confronting Fear of Death*	*	*	*	*
- *Reconciling Guilt*	*		*	*
- *Redefining Communication*	*	*	*	*

Interpretative Phenomenological Analysis was conducted to explore participants’ experiences and psychological challenges raising a child with ASD. The analysis presents two superordinate themes: (i) **from existential crisis to enlightenment**, and (ii) **transcending challenging experiences** (Table 1). These themes capture the essence of the parents’ emotional journeys, with each reflecting aspects of how individuals face unavoidable life challenges that redefine their existence. Jaspers’s concepts of boundary situations [23, 24], existential crises, transcendence, and existential enlightenment are deeply interwoven, with the autistic child as a central catalyst in these parents’ transformative journeys.

### Superordinate theme 1: from existential crisis to enlightenment

This theme reflects the parents’ journey from initial emotional breakdown to gradual acceptance. For all participants, the diagnosis of autism in their child represented a profound boundary situation, reshaping their perceptions of self, human connection, and the future.

### Diagnosis as an existential crisis

For each parent, the diagnosis of autism served as a catalyst for an existential crisis, marking the collapse of previously held assumptions and leading to disorientation. This existential crisis is evident in Anastasia’s account, where she described her experience as a journey through grief:

“I would say it is like a typical journey going through grief. Basically, what you experience feels like something has been taken away, and you don’t know what the future holds… My son was diagnosed when he was 3.5 years old… I cried a lot, and it led to depression.” (Participant 4).

Anastasia’s despair encapsulates the profound shift from her previously envisioned future for her child to an uncertain and uncharted path. Parents often experience intense feelings of loss when confronting the reality that their child may not meet typical developmental milestones [41]. This breakdown also affected the broader family dynamic, as Anastasia reflects on her partner’s experience: “It was a significant mental blow for my son’s father as well, because it felt like the entire world was falling apart.” (Participant 4). The existential crisis extends beyond individual suffering, encompassing a shared collapse of family expectations and dreams.

Similarly, Julia recounted the intense depression and shock she experienced following her child’s diagnosis:

“I was so shocked that I couldn’t sleep that night. I just cried because all my dreams had suddenly been crushed… I was depressed. I didn’t want to wake up. I hated the light, even the mornings. I couldn’t see my future amidst all these problems… It took me almost four months to take everything under control.” (Participant 1).

Julia’s despair, characterised by a retreat from life itself, embodies an existential breakdown where life loses its familiar meaning and direction. Her response demonstrates that parents of children with ASD undergo a period of depression and isolation, as they grapple with the limitations imposed by the diagnosis on their own and their child’s future. Her journey toward acceptance involved an emotional adjustment, navigating depression while confronting the constraints of a new reality.

Peter’s account similarly underscored this rupture:

“It was incredibly shocking to know that someone you love so deeply… will not be able to enjoy their life. It was shattering. It was life changing. I cannot even begin to describe the emotions I felt.” (Participant 3).

His reflection highlights how the diagnosis shatters the assumption of a “normal” life for both parent and child, forcing parents into a state of existential questioning and prompting a re-evaluation of their sense of purpose and their child’s path forward.

Across all accounts, parents initially perceived the diagnosis as a traumatic event marked by shock, a profound sense of loss, overwhelming emotions, grief, and depression. This experience signifies the breakdown of conventional norms and expectations, compelling parents to confront an unforeseen reality. They are faced with the unexpected need to embrace human differences, as the diagnosis suddenly alters their perception of their children, disrupting habitual human connections. This shift underscores the necessity for meaning making and the reconstruction of relational bonds, as parents work toward acceptance and adaptation in the face of an altered reality.

### Acceptance as a form of existential enlightenment

While the initial phase of receiving an autism diagnosis is characterized by grief and crisis, the journey of these parents often leads them toward a form of acceptance. This acceptance is not passive resignation but an active, conscious choice that represents growth and a new orientation toward life. For all participants, acceptance involved acknowledging their child’s unique needs, adapting to new routines, and finding meaning within the reality of their lives.

Marta described this transition poignantly: “It was shocking for me to learn about his diagnosis. It was hard to accept… Then I paused and told myself that he is who he is.” (Participant 2). For Marta, acceptance was tied to maintaining strength for her child: “I try my best to keep my world standing strong for him because if I fall… it won’t be helpful for either of us.” (Participant 2). Her response highlights the existential decision to embrace resilience, not only for herself but as a model of stability for her child. Marta’s words reflect an intentional shift from struggling against the diagnosis to accepting her son’s unique identity, showing resilience and a willingness to let go of previously held expectations. She emphasised the importance of “living in the moment” and advised other parents, “to live day by day and not to have a lot of expectations”. This approach allows Marta to cultivate gratitude for her child’s unique milestones and reframe autism as an opportunity for growth.

Reflecting on her journey, she described it as “a roller coaster of emotions,” adding, “but I always have faith and hope, because I’m a believer.” Her statement suggests that expectations transform into hope through the adoption of spiritual beliefs. Marta spoke about the transformative impact of her son, describing him as “the biggest lesson of my life” and referring to him as “a gift from God” and “an angel.” These descriptions illustrate a spiritual reframing of her experience– one in which boundary situations lead to a deeper understanding of existence, fostering meaningful connection and, ultimately, acceptance.

Peter also acknowledged the active and challenging nature of acceptance: “Accepting the diagnosis is very challenging– understanding your limitations, knowing you cannot just go out and walk on the beach because they have their own routine.” (Participant 3). His reflection underscores the deliberate choice required to adapt to his child’s needs. His account illustrates the shift toward acceptance as an active engagement with his child’s reality, requiring both sacrifice and restructuring his life.

Similar to other participants, Peter found spirituality to be a crucial source of resilience. He described his evolving outlook:

“It’s difficult to talk because the stress is latent. It’s chronic. It stays there with you. You’re sleeping. You’re awake. The best part is you have to believe there are certain things you can do. There’s a lot of things. It’s not in your control. You have to accept that fact. You really have to accept it. You know there’s nothing you can do. However, there are certain things you have to be spiritual about. You have to trust that hopefully things will not be as bad as you are perceiving them to be.” (Participant 3).

Peter’s reflection on his spiritual journey demonstrates how he came to accept the inherent uncertainty and limitations of raising a child with ASD. His practice of hope, centred on acceptance of the present moment, allows him to focus on positive possibilities while remaining grounded in reality. This suggests that spiritual beliefs provide a critical source of strength for parents, helping them navigate the ongoing emotional demands of caregiving.

Hope emerged as a crucial aspect of each parent’s journey, offering a way to navigate the emotional and practical challenges of raising a child with ASD. In boundary situations, hope allows individuals to transcend immediate suffering and find a future-oriented sense of purpose. Julie reflected on hope as an essential component of her resilience:

“Sometimes we give up too easily, but we must believe and stay motivated. You must have this hope inside. If you don’t have hope, it’s a disaster.” (Participant 1).

For Julie, hope functions as a transcendent response to adversity, enabling her to access inner strength and confront life’s challenges with faith and optimism. Her spiritual reframing of her experience as a parent is evident in how she perceives her child:

“When you look into his eyes, you understand that he is just like an angel. He can’t lie. It’s something that you cherish, something divine, like something supernatural in your house.” (Participant 1).

Julie’s use of terms like “supernatural” and “cherish” suggests that her child embodies qualities that transcend ordinary human attributes, positioning him as a profound source of inspiration and meaning. Her reflection conveys a sense of awe and reverence, as she perceives her child not only as a source of love but as a reminder of purity and honesty. This interpretation redefines the parenting experience as more than a caregiving role– it becomes a spiritual journey.

For these parents, the journey from crisis to acceptance is marked by resilience and a reshaped sense of connection. Through this acceptance, parents redefine their identities and find inner strength, ultimately reorienting their lives around their child’s needs. Their experiences reflect a transformative understanding of life, one that transcends personal suffering and reveals the enduring power of human connection.

In each account, the presence of an autistic child serves as a central, transformative force, reshaping the parents’ perceptions of life and meaning. These children catalyse moments of existential crisis, transcendent connection, and spiritual growth, guiding their parents toward new understandings of unconditional love. As they navigate this journey, parents experience a form of existential enlightenment, characterised by deepened appreciation for life’s essential values, rooted in hope, spirituality, and profound human connection. Their children offer powerful lessons in presence and acceptance– ultimately inviting their parents to engage with life on a deeper existential level.

### Superordinate theme 2: transcending challenging experiences

This theme reflects the profound psychological and existential challenges parents face when raising a child with ASD, demonstrating both the enduring worries they carry and the transcendence they experience through this journey.

### Confronting fear of death

All participants reported an acute fear of their own mortality, driven by anxiety over their child’s lifelong dependency. Julia expressed this fear: “I’m still struggling with the fear of leaving my son one day, to die, and to leave him alone. It’s the biggest challenge for me.” (Participant 1). Her words reveal an existential anxiety that goes beyond typical parental worries, reflecting a boundary situation where mortality becomes an unavoidable reality.

Anastasia implicitly expressed this fear:

“I wish there were some support for people who are 18 and older but still on the spectrum. Autism doesn’t disappear. I have some anxiety about it. There is nothing for them– everything falls on the parents’ shoulders. How do you integrate a child into society?” (Participant 4).

This statement highlights the profound fear of mortality and the uncertainty of who will care for the child when parents are no longer around. The absence of societal support for autistic adults exacerbates this fear, forcing parents to confront their own mortality while grappling with the long-term welfare of their children. This aligns with existential concerns about the fragility of life and the responsibility of ensuring stability beyond their own existence.

Peter also reflected on his mortality, noting “The scary part is when I start thinking that something could happen to me. How will my children take care of themselves?” (Participant 3). His words encapsulate the profound vulnerability these parents feel, forced to confront the limitations of control over life’s uncertainties. In response to this fear, Peter mentioned that he and his wife have become more conscious of their health. Such existential awareness reflects boundary situations, where the acceptance of mortality can lead to a heightened appreciation for life and a more intentional approach to well-being.

Marta similarly expressed heightened anxiety: “Normally I’m a person who’s not afraid of death, but because I have him, I’m afraid. That is why I do a medical check-up every year.” (Participant 2), describing how her previously indifferent attitude toward death has shifted since her son’s diagnosis. Her strategy of undergoing regular medical checkups illustrates the lengths to which she goes to prolong her life for her child’s sake– a form of transcendent caregiving that underscores the unique dependency of their relationship.

While this fear of mortality remains ongoing, parents confront it by reframing their focus on the quality of their lives and their health. Their heightened awareness of well-being becomes not only a survival mechanism but also a means of ensuring continued care for their child. By prioritizing their health and well-being, they cultivate a sense of control over an uncertain future and anchor themselves in the present, demonstrating a balance between existential anxiety and meaningful, proactive adaptation.

As all participants’ reflections illustrate, this fear transcends ordinary parental concerns, manifesting as a boundary situation where parents must confront the inherent limits of human control and the uncertainty of their child’s future. For these parents, the awareness of mortality serves as a catalyst for existential transformation, prompting them to find renewed purpose in each moment with their child and take intentional steps to ensure their longevity.

### Reconciling guilt

Guilt permeates parents’ experiences, taking on various forms, including self-blame, comparison with others, and perceived personal failings. Peter’s words illustrated the depth of his internalised guilt:

“Why did it happen? There is this emotional guilt. There is religious guilt, thinking that maybe I’ve done something wrong. People believe in karma. What if I had done something that brought this upon me? You always feel that you could have caused this problem.” (Participant 3).

His reflection on guilt reveals a profound confrontation with the limitations of human control and understanding, situating his experience within existential framework of boundary situations. This exemplifies an existential struggle to make sense of his child’s diagnosis. From an existential perspective, such guilt highlights the human need to find meaning in adversity, even when that search exposes vulnerabilities and uncertainties. This confrontation also presents an opportunity for transcendence, as grappling with guilt may lead to acceptance, helping parents to reframe their role as caregivers within a broader existential context. Intentional conclusions arising from this guilt point to a moral reorientation in parents’ life. This kind of reflection may lead to a heightened commitment to ethical living, emphasising intentional acts of goodness, compassion, and responsibility. Ultimately, this transformative process illustrates the potential for guilt to foster existential transcendence.

Julia discussed her feelings of guilt when comparing her child to others: “His personality is different, completely different. When you compare, you will feel guilty because you don’t see the same results or anything like that.” (Participant 1). This comparison-driven guilt illustrates that parents of children with ASD are prone to self-blame when their child’s developmental progress does not align with societal norms. By reframing her perspective and choosing to view her child as unique, Julia experiences a form of transcendence, acknowledging her child’s individuality and moving beyond societal expectations.

Anastasia’s reflection captured the deep-seated guilt parents often feel when they acknowledge the need for self-care, highlighting an internal struggle to reconcile the demands of caregiving with the need for personal fulfilment. She said:

“You have the right to just be a mom and dad, and you need to make time for yourself as well as a human being. Parents feel guilty. They forget about themselves. It’s probably more severe because there is some isolation and shame.” (Participant 4).

This conflict reveals an underlying guilt, intensified by feelings of “isolation and shame,” suggesting that societal expectations often pressure parents to prioritise their child’s needs to the exclusion of their own. Guilt represents a boundary situation, as parents confront the limitations of their capacities and face the impossible standard of complete self-sacrifice. The guilt experienced here is not merely personal but existential, reflecting a struggle with self-worth and identity, as these parents attempt to retain their humanity amid overwhelming caregiving responsibilities. The acknowledgment that “parents forget about themselves” highlights the depth of this sacrifice, where guilt and isolation become intertwined, potentially leading to emotional burnout. This internal conflict may ultimately drive parents toward an existential transformation, where they must redefine their sense of self and caregiving to preserve both their own well-being and the relationship with their child.

### Redefining communication

Raising a child with autism redefines the meaning of communication for all these parents, fostering a transcendent form of connection that goes beyond verbal exchanges. Transcendence involves moving beyond ordinary experiences to connect with something deeper, and for these parents, their children serve as profound teachers, helping them discover new ways of expressing and understanding love, patience, and empathy.

Anastasia’s reflection captured this complexity:

“I’m getting worried, fantasizing that somebody can be mean to him, or some bullies might bother him. So, when he tells me about stuff, I feel very excited, because even though he is fully verbal, I still feel this lack of communication.” (Participant 4).

This statement reflects the complexity of verbal ability in autism, where speech does not necessarily equate to reciprocal, emotionally fulfilling communication. Anastasia’s sense of “excitement” indicates how even brief communicative exchanges feel profoundly meaningful to her. Her response highlights an existential appreciation for these moments, embodying the notion of transcending limitations to foster a deeper understanding and acceptance of her child’s unique way of relating to the world.

Marta also described her son’s unique communication style, saying, “Sometimes I feel like I’m talking to a wall… He does not respond to me immediately, but he responds at his own pace.” (Participant 2). While initially challenging, Marta comes to appreciate her son’s distinct way of interacting, finding value in the diversity of human expression. This shift indicates that parents of children with ASD often reach a point of acceptance and appreciation for their child’s unique forms of communication, redefining their understanding of connection.

For Peter, his children’s limited verbal expressions are still deeply meaningful and fulfilling. He explained:

“Honestly, it’s extremely, extremely tough. No matter what kind of support you have, the fact that you come home, and you wave to your son who has autism, and he cannot acknowledge, is difficult. They cannot reciprocate…. You know, you don’t lead a normal life… but when my son smiles, trying to say ‘Daddy,’ it’s priceless. It makes me forget everything in that moment.” (Participant 3).

In these small, nonverbal connections, Peter experiences the “depth” dimension of life– a connection that surpasses conventional measures of success or societal norms. This transcendent understanding of love demonstrates that parents of children with autism may redefine happiness and fulfilment based on unique, non-traditional connections with their children.

For all these parents, the nonverbal or limited verbal expressions of affection from their children become deeply meaningful. Julie described how her son’s simple yet repetitive expression of love, “He says ‘I love you’… 100 times a day, but we are not bored” (Participant 1), carried profound significance, as she interpreted this as a genuine connection and a “best moment” that surpasses ordinary expressions of affection. Through their children, parents encounter new depths of love and connection that transcend verbal communication. These children become profound teachers, guiding their parents to discover the beauty in nonverbal connections, small milestones, and the unique ways that love and patience are expressed.

## Discussion

This study explored the lived experiences of parents raising children with ASD, focusing on their emotional challenges. By adopting an existential perspective within the framework of IPA, this research addressed a gap in existing literature by interpreting how parents of children with ASD develop an existential approach to confront the adversities associated with ASD and adapt to life with renewed meaning and resilience. The findings illuminate how these parents navigate boundary situations, including existential crisis, acceptance, fear of death, guilt, and communication challenges. These challenges ultimately culminate in spirituality and profound changes in parents’ sense of self, purpose, and connection. The findings of this study align with and expand on the theoretical framework presented in the introduction, demonstrating how existential philosophy and concepts such as boundary situations, transcendence, and existential transformation manifest across superordinate themes– From existential crisis to enlightenment and Transcending challenging experiences.

The **From existential crisis to enlightenment** theme highlights how the diagnosis of ASD functions as a profound boundary situation, disrupting previously held assumptions about parenting, the future, and expectations. Participants’ initial reactions of grief, despair, and disorientation resonate with Jaspers’s [23] concept of boundary situations as unavoidable confrontations with existential realities. This echoes findings by Gray [41], who observed that parents of children with ASD frequently undergo a period of depression and isolation, as they grapple with the limitations imposed by the diagnosis on their own and their child’s future. The findings reveal that this *existential crisis* is characterised by emotional breakdowns and a re-evaluation of self and purpose, aligning with the distress-growth duality identified by Phelps et al. [22]. The journey toward *acceptance* emerges as an existential enlightenment, wherein parents actively reorient their perspectives and embrace their child’s unique needs. Participants’ reflections on adapting to new routines and redefining family expectations underscore the transformative potential of acceptance. This transformation resonates with the neurodiversity acceptance movement [42], suggesting that the act of acceptance extends beyond the individual level to challenge societal norms, fostering greater inclusivity, empathy, and understanding of neurodivergence. Acceptance as an existential decision aligns with the findings of Waizbard-Bartov et al. [26], emphasising the active role parents play in reconstructing their lives around their child’s individuality. It also echoes findings by Da Paz et al. [43], who identified that parents of children with ASD often undergo a period of redefining expectations, finding ways to accept the challenges posed by the diagnosis. This process illustrates the resilience and agency required to confront boundary situations and underscores the importance of meaning-making in fostering psychological and emotional adaptation. Acceptance is fostered through the adaptation of spiritual beliefs, where hope and faith emerge as crucial components of existential transformation. Participants reframed their parenting experiences as spiritual journeys, perceiving their children as sources of divine inspiration. These findings align with research by Ekas et al. [25] and Waizbard-Bartov et al. [26], which highlight the role of spirituality in fostering resilience and well-being among parents of children with ASD.

The **Transcending challenging experiences** theme revealed the intense emotional challenges parents face, including *fear of death*, *guilt* and *dissatisfaction with communication*, and their potential for transformative growth. *Fear of death* emerged as a profound existential concern among participants, centred on the long-term welfare of their children in the absence of structured societal support. Parents reflected on their mortality and described proactive measures to prolong their lives and concentrate on the present moment. These experiences align with Jaspers’s [23] view that confrontations with finitude can evoke a heightened appreciation of the present moment. The participants’ reflections highlight the dual nature of this fear: while deeply distressing, it serves as a motivator to live purposefully. This finding underscores the importance of interventions that address both the practical and emotional dimensions of this fear. Spiritual and mindfulness practices, which encourage a focus on the present moment, have shown positive effects on resilience among parents of children with ASD [27], further supporting the potential for such approaches.

Much like fear, *guilt* was identified as an existential struggle to derive meaning from adversity. Participants reframed guilt in transformative ways. For instance, guilt stemming from comparing their children to others helped parents recognise and accept diversity. This process reflects Heidegger’s [35] notion of *being-with-others*, emphasising that accepting the differences in others fosters deeper connections and shared humanity. Acceptance of diversity enabled participants to cultivate a sense of coexistence with difference, balancing their own needs with those of their children. Similarly, guilt linked to self-blame and the search for causes of ASD encouraged participants to strive for morally grounded lives, reframing their guilt as a catalyst for growth. This transformation aligns with Sartre’s [29] concept of *existentialism as a humanism*, which posits that human beings create their essence through their actions and decisions, particularly in response to life’s challenges. By confronting guilt and reshaping their understanding of themselves and their roles as caregivers, participants actively constructed new meanings and values in their lives.

The *Redefining communication* subordinate theme explores the broader transformative experiences of parents as they redefine communication. This theme reflects Jaspers’s [24] concept of transcendence, wherein individuals move beyond conventional norms to derive new meanings from their lived experiences. Participants described reimagining communication as a deeply emotional and often nonverbal connection with their children. Their accounts of cherishing their children’s unique expressions illustrate how transcendence involves embracing differences and finding joy in small yet profound moments of connection. This aligns with findings by Tager-Flusberg et al. [44] on the complexities of verbal communication in ASD and underscores the importance of fostering attuned, nonverbal interactions as pathways to meaningful relationships.

Thus, the study suggests that the existential and experiential transformation of parents raising children with ASD consists of the following key components:

- *Collapse of previously held expectations*– Parents experience a profound disruption in their assumptions about parenting, navigating despair and grief as they adjust to their child’s diagnosis.
- *Transcendence of norms and re-evaluation of meaning*– Through confronting intense emotions such as fear of death, guilt, and dissatisfaction with communication, parents reassess their perspectives on life and caregiving.
- *Acceptance of differences and the development of shared humanity*– Embracing their child’s uniqueness fosters unconditional love, compassion, and a deeper sense of human connection.
- *Transition from despair to hope through spiritual beliefs*– The adoption of spirituality and faith serves as a catalyst for resilience, enabling parents to find hope and meaning in their journey.

This transformation reflects an ongoing existential journey, wherein parents move from crisis to acceptance, ultimately discovering growth, connection, and purpose in their evolving roles.

### Strengths, limitations and future directions

By employing IPA, this study offered a nuanced understanding of parents’ lived experiences. The integration of an existential perspective deepened the analysis, emphasising the transformative potential of confronting life’s limitations and fostering resilience. The study underscores the interplay between distress and growth, revealing how parents navigate challenging emotions to achieve acceptance and personal growth.

While the idiographic nature of IPA supports the use of a small sample, the findings may not generalise to wider populations. Future research should consider larger, more diverse samples to capture a broader range of experiences. The sample included three mothers and one father, reflecting a common trend in caregiving research where mothers are more likely to volunteer for studies involving emotional reflection. While not a criterion for selection, this gender imbalance may limit the diversity of parental perspectives captured in the findings. Additionally, participants who volunteered for this study may have been more inclined to reflect on their experiences, potentially biasing the data toward those with higher self-awareness. However, this also highlights the potential for transformation and growth among parents who struggle with mental health challenges because of caregiving for children with ASD.

Another limitation of the study is the lack of detailed demographic data beyond age, gender, country of residence, and parental role. While this decision aligned with the idiographic nature of IPA, which prioritizes depth over breadth, it may limit the contextual richness that additional variables (e.g., socioeconomic status, education level, marital status) could have provided. Future research may benefit from collecting more comprehensive demographic profiles to explore how intersecting factors shape the lived experiences and adaptive processes of parents raising children with ASD.

While the inclusion of participants from four different countries (Canada, the United Arab Emirates, the United Kingdom, and the United States) added cultural and contextual diversity to the data, this also presents a methodological limitation. With only one participant from each country and no detailed information about their cultural backgrounds, healthcare systems, or ASD-related support structures, it is difficult to determine the extent to which the identified themes reflect universally shared parental experiences versus those shaped by specific cultural contexts. Given the existential and meaning-making orientation of this study, which emphasizes the universality of emotions such as grief, guilt, hope, and transformation, cultural identity was not a central focus. However, future research should further investigate how sociocultural frameworks influence the interpretation of existential experiences and the ways in which parents adapt to their child’s ASD diagnosis.

Future research may build on these findings using a Convergent Parallel Design, integrating qualitative (IPA interviews) and quantitative (psychological surveys) to enhance understanding. Parents would complete validated measures assessing stress, depression, existential anxiety, post-traumatic growth, coping strategies, well-being, spirituality, and resilience, while interviews explore narratives of transformation and adaptation. By analysing both datasets separately and then integrating the findings, researchers can examine whether survey data align with participants’ lived experiences in interviews. This triangulation strengthens validity and offers deeper insights for developing targeted interventions to support parents navigating the complexities of raising a child with ASD.

The findings highlight the importance of interventions addressing existential and spiritual dimensions, fostering resilience, acceptance, and well-being among parents. Future research should explore intervention-based approaches using an Embedded Design, integrating quantitative and qualitative methods to evaluate the effectiveness of targeted support programs for parents of children with ASD. One potential direction is implementing parental interventions such as mindfulness therapy, existential therapy, or peer support groups, aimed at helping parents navigate stress, existential anxiety, and meaning making in their caregiving journey. Another approach is to develop intervention programs tailored to the specific emotional challenges identified in this study. Pre- and post-intervention psychological measures (e.g., stress reduction, resilience, well-being scales) would quantify improvements in mental health and coping strategies, while IPA interviews would provide insights into parents’ subjective experiences of transformation. This approach directly tests practical solutions, ensuring both measurable impact and rich narrative insights into how interventions facilitate parental adaptation. By integrating empirical assessment with lived experiences, this design could inform the development of evidence-based support strategies.

Exploring the perspectives of autistic children within the family dynamic could provide complementary insights, particularly regarding how parental attitudes influence children’s emotional and behavioural outcomes.

## Conclusion

This study contributes to the growing body of research on the lived experiences of parents raising children with ASD by emphasising the existential and spiritual dimensions of their journey. The findings reveal that parenting a child with ASD is not only a profound challenge but also an opportunity for growth and existential transformation. Through an existential framework, the study illustrates how parents confront crises, transcend emotional challenges, and forge deeper connections with their children. These insights offer a foundation for interventions that promote resilience, acceptance, and spiritual well-being, ultimately empowering parents in their transformative journey.

## Electronic supplementary material

Below is the link to the electronic supplementary material.

Supplementary Material 1

Supplementary Material 2

Supplementary Material 3

## Data Availability

Data is provided within the manuscript. While extracts from discussions have been shared in the results section of this research paper, we will not be sharing full transcripts.

## Abbreviations

ASD: Autism Spectrum Disorder
IPA: Interpretative Phenomenological Analysis
DSM-5: Diagnostic and Statistical Manual of Mental Disorders, Fifth Edition
