# Supplementary material for: “Like something supernatural in your house”: an interpretative phenomenological analysis to explore the experiences and psychological challenges of parents raising children with autism spectrum disorder
Source: BMC Psychol. 2025 Jul 1;13:642. doi: 10.1186/s40359-025-03057-5 (PMC12210484; doi:10.1186/s40359-025-03057-5)
Supplement: Supplementary file 1 — Supplementary Material 1 [file 40359_2025_3057_MOESM1_ESM.docx]

**Semi-Structured Interview Guide**

An interpretative phenomenological analysis to explore the lived experiences of parents raising children diagnosed with ASD

1. Can you describe your emotional journey since your child was diagnosed with ASD?

- Follow-up: How have your feelings and emotions evolved over time?

2. What have been the most significant challenges you have faced as a parent of a child with ASD?

- Follow-up: Can you provide specific examples of these challenges?

3. How do you typically cope with any stress associated with raising a child with ASD?

- Follow-up: What strategies have you found most and least effective?

4. Can you tell me about your support system? Who do you rely on for support and how do they help you?

- Follow-up: How has your support system impacted your well-being?

5. How would you describe your relationship with your child?

- Follow-up: What activities or moments do you find most rewarding and why?

6. In what ways have your interactions and communication with your child been affected by their ASD diagnosis?

- Follow-up: Have you developed any specific strategies or approaches to improve communication?

7. How has raising a child with ASD impacted your personal life, such as your career, social relationships, or personal time?

- Follow-up: What changes have you made to accommodate your child's needs?

8. What emotions do you commonly experience in relation to your child's ASD, and how do you manage these emotions?

- Follow-up: Are there any specific triggers that intensify these emotions?

9. Can you discuss any specific instances where you felt overwhelmed or struggled to cope with your child's behavior?

- Follow-up: How did you handle those situations, and what support did you seek?

10. What advice would you give to other parents who are just starting their journey with a child diagnosed with ASD?

- Follow-up: What resources or strategies have been most helpful to you?

11. Is there anything else that you would like to mention that we haven’t discussed?
