# Supplementary material for: “Like something supernatural in your house”: an interpretative phenomenological analysis to explore the experiences and psychological challenges of parents raising children with autism spectrum disorder
Source: BMC Psychol. 2025 Jul 1;13:642. doi: 10.1186/s40359-025-03057-5 (PMC12210484; doi:10.1186/s40359-025-03057-5)
Supplement: Supplementary file 3 — Supplementary Material 3 [file 40359_2025_3057_MOESM3_ESM.docx]

**Point-by-Point Response to Reviewers’ Comments**

Manuscript Title:
“Like Something Supernatural in Your House”: An Interpretative Phenomenological Analysis to Explore the Experiences and Psychological Challenges of Parents Raising Children with Autism Spectrum Disorder

**Reviewer 1**

This manuscript explores the lived experiences of parents raising children with ASD using an existential framework and Interpretative Phenomenological Analysis (IPA). The topic is relevant and emotionally resonant, and the manuscript provides in-depth qualitative insights into an under-researched dimension of caregiving: existential transformation. However, the manuscript requires major revisions in methodological transparency and clarity to improve its academic rigor and coherence.

**1. Introduction**

The introduction builds a compelling rationale for exploring existential dimensions in ASD parenting. However:

**Comment:**

While citations are extensive and up-to-date, some claims (e.g., “acceptance promotes attunement”) would benefit from more direct linkage between the claim and its supporting citation. In particular, several assertions in the second paragraph on page 5 require exemplifying references to enhance their credibility.

**Response:**

We have revised the introduction, including the second paragraph on page 5 to strengthen the empirical grounding of the claims presented. Specifically, we added references that support the role of spirituality as a coping resource for parents of children with ASD, and we justified the choice of IPA. (Page 4, line 89; page 5, line 111)

**Comment:**

The following statement also requires supporting and exemplifying citations: “In summary, while existing research has shed light on the psychological difficulties faced by parents of children with ASD, it often lacks a comprehensive understanding of how they adapt to these challenges.” To strengthen this claim, I recommend citing specific studies that highlight this gap in the literature, particularly those that focus predominantly on parental stress, depression, or burden without exploring meaning-making, existential adaptation, or post-traumatic growth.

**Response:**

We revised the paragraph to better articulate the gap in the literature on parental adaptation by adding citations that illustrate the focus on stress, depression, and burden. We also included citations to acknowledge emerging research on post-traumatic growth, while noting that the processes behind such growth remain under-explored. The revised text highlights the need for further research into meaning-making and existential adaptation, and clarifies how our study addresses this gap through an existential framework. (Page 6, line 141)

**3. Method**

The method section is largely sound, though a few areas need clarification:

**Comment:**

The authors report that participants reside in four different countries but do not provide their nationalities or cultural backgrounds. Given that cultural values shape existential interpretation and parenting norms, this omission limits the reader’s ability to contextualize the lived experiences shared in the study.

**Response:**

We did not gather detailed information about participants’ nationalities or cultural backgrounds. This choice was consistent with the existential-phenomenological orientation of the study, which focuses on the universality of emotional and existential experiences. We sought to understand how parents make sense of profound emotional challenges that transcend cultural boundaries, such as fear, loss, guilt, hope, and growth. However, we recognize that cultural values can shape how these experiences are interpreted and expressed. We have therefore addressed this nuance in the revised “Strengths, Limitations and Future Directions” section. (Page 31, line 748)

**Comment:**

Although two authors are listed on the manuscript, the Analytical Strategy section consistently refers to "the researcher" in the singular, implying that only one person conducted the data analysis and interpretative work. This raises questions about the specific contributions of the second author and whether they were involved in the analytic process, interpretation, or oversight. I recommend clarifying in the methods section who conducted the analysis and how responsibilities were distributed between co-authors. This clarification is especially important given the interpretative and reflexive nature of IPA, which relies heavily on the subjective lens of the analyst.

**Response:**

We have revised the Analytical Strategy section to clarify the roles of both authors in the analysis process. The first author conducted the core IPA analysis following the six-step approach, while the second author reviewed transcripts, quotations, and the narrative structure of the results to ensure interpretative accuracy and coherence. This clarification strengthens transparency and addresses the reflexive and interpretative requirements of IPA. (Page 11, line 265)

**4. Results**

The findings are rich, insightful, and emotionally evocative.

**5. Discussion**

The thoughtful and expansive discussion connects findings to existential theory and relevant literature.

**6. Strengths and Limitations Section**

**Comment:**

I recommend explicitly including the geographic diversity of the sample as a limitation in the Limitations section. For example, the authors could write:

“While the inclusion of participants from four different countries (Canada, the UAE, the UK, and the US) added cultural and contextual diversity to the data, this also presents a methodological limitation. With only one participant representing each country and no in-depth exploration of specific cultural norms, healthcare systems, or national ASD-related support structures, it is difficult to determine the extent to which the themes identified reflect universal parental experiences versus culturally shaped ones. Given the existential and meaning-making focus of the study, future research should explore how sociocultural context may influence the ways in which parents interpret and adapt to their child’s ASD diagnosis.”

**Response:**

We acknowledge that the absence of detailed information about participants’ nationalities and cultural backgrounds limits the ability to fully contextualize the findings in relation to cultural norms and healthcare systems. This limitation has now been explicitly addressed in the “Strengths, Limitations and Future Directions” section. While the existential-phenomenological orientation of the study emphasizes universal emotional and existential themes, we agree that future research would benefit from examining how sociocultural factors influence parental meaning-making and adaptation processes. (Page 31, line 748)

**Reviewer 2**

Thank you for giving me the opportunity to review this article. The article addresses a very important topic for few reasons. Parents’ weal-being reflect largely on their autistic children. To give parents voice to share their personal experiences, their emotions and how they reach resilience is an important part about autism. Therefore, I recommend for the paper to be published with the following considerations.

**General Observations**

Although the authors explained the part about the (diversity of the participants) and the number of participants based on the IPA method, still I believe having such a big gap in the participants’ age, and similarly in the autos’ age in addition to the cultural differences (UAE, UK, USA), can affect the results of the research. Emotions about raising an autistic child and reaching resilience vary deeply depending on age of the parent/child and their gender, and to address these issues with such a diverse group may need to be justified. For example, a question like “Can you describe your emotional journey since your child was diagnosed with ASD?” would have a very different response if the autistic child is 11 and/or if they are 22. Further, a mother’s experience being the usual primary caretaker is different from the father’s experience.

**Response**

Thank you for this valuable observation. We appreciate the importance of demographic variation in shaping individual experiences. Rather than aiming for demographic representativeness or control, the study sought to explore how different individuals make sense of emotionally intense and existentially challenging experiences related to parenting a child with ASD. In IPA, variation in participant characteristics is not seen as a confound but as a potential source of richness, helping illuminate both unique and shared meaning-making processes. While age, gender, and cultural background undoubtedly influence emotional responses, our analysis focused on the underlying experiential structures that cut across such differences. That said, we acknowledge the reviewer’s point and agree that future research with more demographically homogeneous or intentionally stratified samples may further clarify how specific factors shape adaptation and emotional journeys.

**Recommendations**

1. I suggest adding the language of the interviews, English/ Arabic specially that the authors have participants from United Arab Emirates and linguistic (exploring the language and expression used) was taken into consideration.

**Response**

All interviews were conducted in English, and fluency in English was one of the inclusion criteria for participation. As such, linguistic analysis was conducted in English, and no translation was required. We acknowledge the importance of clarifying this in the manuscript and have now added a statement to the Method section under “Procedure” to ensure transparency regarding interview language and participant eligibility. (Page 10, line 233)

2. I suggest adding more information about the online interviews, were they recorded, was the permission to record obtained? Audio, video.. etc..

**Response**

All interviews were conducted via Microsoft Teams and were both audio- and video-recorded with participants’ informed consent. Participants received an information sheet and completed a signed consent form prior to the interview, which explicitly included permission for audio and video recording for research purposes. This clarification has been added to the “Procedure” subsection in the Method section of the manuscript. (Page 10, line 235)

3. I suggest adding additional demographic information about the participants.

**Response**

We acknowledge the value of including more detailed demographic data to further contextualize participants' experiences. However, beyond the information already provided in the study, no additional demographic data were collected. This decision was made to maintain a focused, in-depth exploration of lived experiences within the idiographic framework of IPA. We have addressed the limitations of this approach in the “Strengths, Limitations and Future Directions” section and agree that future research may benefit from a broader demographic profile. (Page 30, line 741)

4. How was the identity of the participants protected?

**Response**

All participants were assigned pseudonyms during transcription, and any potentially identifying information was removed or altered to maintain confidentiality. Audio and video recordings were stored securely and deleted following transcription. Transcripts were anonymized and stored on a password-protected institutional account. These procedures ensured that participant identities remained protected throughout the research process. A clarifying sentence has been added to the “Ethical Considerations” subsection of the Method section. (Page 13, line 311)

5. The participants’ words were followed by numbers such as (Participant 4), it might be better to perhaps add the nationality of the participants.

**Response**

While we agree that including participant nationality can provide valuable context, we did not collect information on participants’ nationalities during data collection. This decision was consistent with the existential-phenomenological orientation of the study, which emphasises the universality of emotional and existential experiences across cultural contexts. However, we acknowledge that the absence of cultural background data may limit the reader’s ability to fully contextualise the findings. We have addressed this as a limitation in the “Strengths, Limitations and Future Directions” section. (Page 30, line 741)

6. Is there a reason that 3 mothers and 1 father were chosen?

**Response**

Thank you for this important question. Participants were not selected based on gender; rather, they self-selected in response to the study invitation. The resulting sample of three mothers and one father reflects a common pattern in caregiving research, where mothers are more likely to volunteer for studies involving emotional reflection on parenting. This gender distribution was not intentional but emerged naturally through the recruitment process. We have now acknowledged this in the “Strengths, Limitations and Future Directions” section as a potential limitation regarding the diversity of parental perspectives. (Page 30, line 732)

7. End of page (14), when state that… Parents often experience intense feelings…, I suggest adding a reference.

**Response**

We located the statement in the Results section under the “Diagnosis as an existential crisis” theme and agree that additional referencing strengthens this claim. We have now added a reference to support the observation that parents often experience intense feelings of loss when faced with their child’s diagnosis. (Page 15, line 377)
